# Supplementary material for: Antenatal Ureaplasma Infection Causes Colonic Mucus Barrier Defects: Implications for Intestinal Pathologies
Source: Int J Mol Sci. 2024 Apr 3;25(7):4000. doi: 10.3390/ijms25074000 (PMC11011967; doi:10.3390/ijms25074000)
Supplement: Supplementary file 1 [file ijms-25-04000-s001.zip › ijms-2918073-supplementary.pdf]

Supplementary Material

Supplementary Table S1 Antibodies used

**Primary antibodies used:**
polyclonal rabbit anti-mucin 2 (MUC2c3)<br>kindly provided by Gunnar Hansson, University of Gothenburg, Gothenburg, Sweden
polyclonal rabbit anti-immature mucin 2 (PH 489)<br>kindly provided by Gunnar Hansson, University of Gothenburg, Gothenburg, Sweden
polyclonal rabbit anti-SPDEF<br>(ab197375, Abcam, Cambridge, United Kingdom)
monoclonal rabbit anti-BiP<br>(C50B12 #3177 Cell Signaling Technology, Danvers, MA, USA)
monoclonal mouse anti-CHOP<br>(9C8, MA1-250, Invitrogen, Carlsbad, CA, USA)
**Secondary antibodies used:**
biotin-conjugated polyclonal swine anti-rabbit<br>(E0353, DakoCytomation, Glostrup, Denmark) (MUC2, iMUC2, SPDEF. BIP)
biotin-conjugated polyclonal rabbit anti-mouse<br>(E0413, DakoCytomation, Glostrup, Denmark) (CHOP)

Supplementary Table S2 Sequences of primers used

**Primer**	**Forward**	**Reverse**
**Housekeeping genes**		
ovRPS15	5′-CGAGATGGTGGGCAGCAT-3′	5′-GCTTGATTTCCACCTGGTTGA-3′
GAPDH	5′-ATGCCTCCTGCACCACCA-3′	5′-ATGCCCTCCACGATGCCAA-3′
YWHAZ	5′-TGAACTCCCCTGAGAAAGCC-3′	5′-TCCGATGTCCACAATGTCAAGT-3′
**Genes of interest**		
GCLC	5′- CCCGATGAAGCCATCAACAAG-3′	5′- TCTTGTCCTTAAAGATGGGCACG-3′
HMOX1	5′- CCGCCTTCCTGCTCAACATC-3′	5′- GGGAGCCGAGTCTTGTGCC-3′
TXNRD1	5′- TGGGGAAGAAAATGTTGAGGTTT-3′	5′- GACCCAGTATGTGGAAGCCC-3′

Abbreviations: ovRPS15: ovine ribosomal protein S15, GAPDH, glyceraldehyde 3-phosphate dehydrogenase, YWHAZ: 14-3-3 protein zeta/delta, GCLC: glutamate-cysteine ligase catalytic subunit, HMOX1: heme oxygenase 1; TXNRD1: thioredoxin reductase 1.

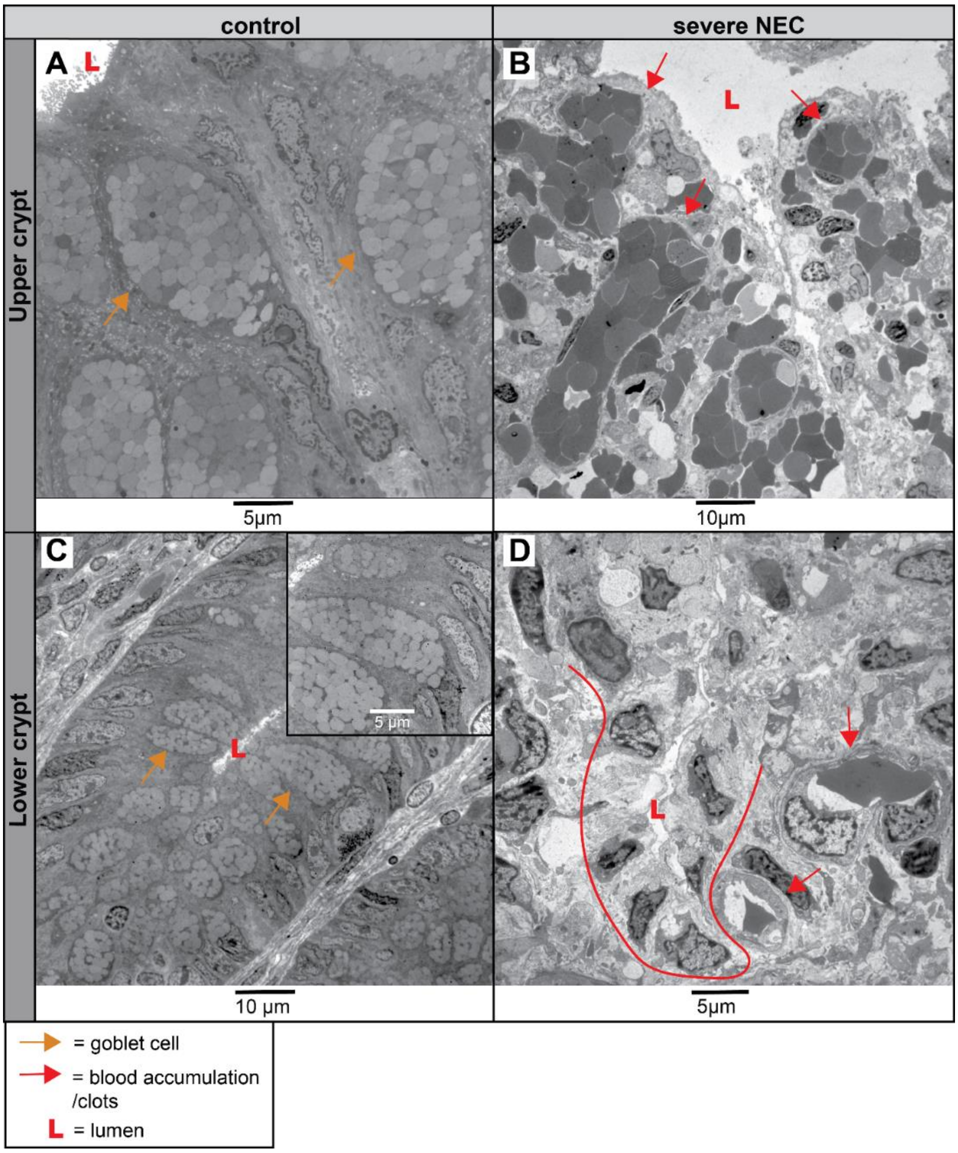

Supplementary Figure S1. Cellular morphology and tissue organisation of colonic biopsies of a NEC patient with severe damage and controls imaged with TEM. Whereas the upper crypt of control infants consists of organized and identifiable goblet cells and colonocytes (A, N=2), the tissue organization and cellular morphology of the upper colonic crypt in the NEC patient is completely and severely damaged; mainly blood accumulation and infiltration (red arrows) is seen in this severely injured and necrotic tissue (B, N=1). In the lower crypt of controls, the organization and morphology of both colonocytes and goblet cells (insert) are normal (C, N=2). The tissue and cellular organization is completely disrupted in the NEC patient; no recognizable lower crypt structure (red line) or cellular morphology is observed; small capillaries (red arrows) are present (D, N=1). Scale bars indicate 5µm and 10µm respectively. Abbreviations: GA: gestational age; L: lumen; NEC: necrotizing enterocolitis; TEM: transmission electron microscopy
